# Liver injury with COVID-19: laboratory and histopathological outcome—systematic review and meta-analysis

**DOI:** 10.1186/s43066-022-00171-6

**Published:** 2022-01-21

**Authors:** Sherine A. Mohammed, Khalid M. Eid, Felix Emeka Anyiam, Hazem Wadaaallah, Muhamed Ahmed Mahmoud Muhamed, Maha Hosni Morsi, Nesrine Ben Hadj Dahman

**Affiliations:** 1 Faculty of Medicine, Sohag University, Sohag, Egypt; 2 Faculty of Pharmacy, Al-Azhar University, Assiut Branch, Assiut, Egypt; 3 Centre for Health and Development, University of Port Harcourt, Port Harcourt, Nigeria; 4 Biomedical Physics Department, Faculty of Science, Helwan University, Cairo, Egypt; 5 Faculty of Medicine, Minya University, Minya, Egypt; 6 Misr University for Science and Technology, 6th of October City, Egypt; 7 Faculty of Medicine of Tunis, University of Tunis El Manar, Tunis, Tunisia

**Keywords:** COVID-19, Abnormal liver function, Autopsy, Liver function tests

## Abstract

**Background:**

The novel coronavirus severe acute respiratory syndrome coronavirus 2 (SARS-CoV-2) infection has been predominantly linked to respiratory distress syndrome, but hepatic injury has also been reported. The mechanism of liver injury is poorly understood.

This review aimed to systematically review the current data through laboratory tests and liver tissue pathology to ascertain the correlation of liver involvement in SARS-CoV-2 infection patients.

**Methods:**

The PubMed, Scopus, Science Direct, and Web of Science databases were searched systematically. We included peer-reviewed published papers available online as clinical cases, cohort studies, and retrospective studies, for both in vitro and in vivo human studies. Independent extraction of the data was done by two independent authors.

**Results:**

A total of 15 articles were finally included in the systematic review process and meta-analysis after exclusion of studies that did not meet the eligibility criteria, summarized in a PRISMA flow diagram.

The meta-analysis showed that patients with underlying abnormal liver function and/or histopathological finding had a statistically significant 8.08 times higher odds of severe COVID-19 outcomes when data from the individual studies were pooled (*OR* 8.08; 95% *CI*,3.43, 19.03; *p* = 0.00001). Five of these studies showed histopathological changes on autopsy from cases with severe COVID-19, and in four of these five studies, the histopathology was associated with a history of abnormal liver function after affection with COVID-19.

**Short conclusion:**

The study observed that the severity of COVID-19 was associated with more patients with aberrant liver function tests.

## Background

Coronaviruses represent a group of viruses that incline to infect the upper respiratory tract, causing moderate to severe illnesses, ranging from the typical cold to pneumonia in severe cases [1].

Recurrent gastrointestinal symptoms were observed in COVID-19 patients, which were late-stage symptoms associated with an increase in the severity of disease [2].

A previous study on the SARS virus demonstrated that more than 50% of patients showed varying hepatopathy levels, particularly in the form of elevated liver enzymes [3]. Another study recorded a high pervasiveness of abnormal aminotransferase levels in severe COVID-19 patients, which may be of non-hepatic origin [4]. Notably, increased alanine aminotransferase (ALT) activity has been reported to be a significant attribute of severe/critical COVID-19 [5]. Abnormal liver tests may be associated with the virus or the drug therapy. The hepatic injury detected in the early phase of the viral disease may culminate in an immediate insult of the virus or incubation of a part of the infection-associated complex systemic inflammatory response syndrome (SIRS) [6]. A number of blood tests are available that reflect the condition of the liver. The most common tests used in clinical practice include the serum aminotransferases, bilirubin, alkaline phosphatase, albumin, and prothrombin time. These tests are often referred to as “liver function tests,” although this term is somewhat misleading since most do not accurately reflect how well the liver is functioning, and abnormal values can be caused by diseases unrelated to the liver. In addition, these tests may be normal in patients who have advanced liver disease; in COVID, we stated that liver enzymes are abnormal and we discussed that in the “Discussion” section.

Our goal is to systematically review current data through laboratory tests and liver tissue pathology to confirm the correlation of liver involvement in SARS-CoV-2 infection patients and detect the prognosis relationship with COVID-19 patients.

## Main text

### Methods

We performed this review and meta-analysis according to the Preferred Reporting Items for Systematic Reviews and Meta-analysis (PRISMA) guidelines and the Cochrane Handbook for Systematic Reviews of Interventions [7].

### Literature search strategy

We performed a comprehensive search on PubMed, Scopus, Science Direct, and Web of Science databases using the following search queries: Monitoring OR assessment AND (Hepatic damage) OR liver histopathology OR (Covid-19 and Hepatic Injury) OR (Hepatic cell failure) AND Covid19 OR Covid-19 OR Novel-Coronavirus OR Severe Acute Respiratory Distress Syndrome Coronavirus 2 And 2019-n COV, SARS-COV-2.

### Inclusion and exclusion criteria

We have included published peer-reviewed papers available online with the following criteria:

(1) Clinical cases, cohort studies, retrospective studies, and in vitro and in vivo human studies; (2) studies providing sufficient reliable data to aggregate in a meta-analysis; and (3) articles in all languages in 2020. (4) Reported outcome data on the risk severity of liver injury between severe and non-severe COVID-19.

In the case of multiple reports for the same study population, we analyzed data of a complete dataset. Studies were excluded for the following reasons: (1) review articles and (2) incomplete data.

### Study selection

Two authors (Khalid M. Eid and Muhamed Mahmoud) applied the selection criteria. The eligibility screening was performed in two steps: the first step was to screen abstracts for eligibility, whereas in the second step, full-text articles of eligible abstracts were retrieved and screened for eligibility to meta-analysis.

### Data extraction

Three authors (Khalid M.Eid and Sherine A. Elsherif) independently extracted the data on an Excel sheet under the supervision of Nesrine Ben Hadj Dahman. The following data were extracted: (1) study design characterization (study design type, population, sample size, and main outcomes) and (2) the baseline characteristics of the included studies (location, group, sample size, age, gender, intervention, treatment duration, and primary clinical diagnosis).

### Quality assessment

It was performed by two authors (Hazem Wadaallah, Muhamed Mahmoud) under the supervision of Sherine A. Elsherif and Nesrine Ben Hadj Dahman. We used quality assessment via the modified Newcastle-Ottawa scale for cohort studies [8]. The scoring consists of 8 questions: representation of the average adult in the community (population-based study = 1 point ; multicenter = 0.5 point; single center = 0 point), cohort size (more than 100 subjects = 1 point, between 50 and 99 subjects = 0.5 point, less than 50 subjects = 0 point), reported information on percentages and pattern of liver injury (information with clarity = 1 point, information derived from percentages = 0.5 point, unclear = 0), reported percentages of subjects with chronic liver disease at baseline (yes = 1 point, no = 0 point), assessed factors between mild and severe COVID-19 (yes = 1, no = 0), adequate clinical assessment (yes = 1, no = 0), sufficient follow-up period for outcome to occur (yes = 1 point, unclear = 0 point), and adequate follow-up (all subjects were followed up = 1 point, > 50% subjects were followed up = 0.5 points, < 50% subjects were followed up = 0 point). We considered studies with a score of ≥6, 3–4, and < 3 to be high-quality, medium-quality, and low-quality, respectively.

### Statistical analysis

It was performed by Felix Emeka Anyiam. Statistical heterogeneity was assessed by visual inspection of forest plots and measured by *I*^2^ and *χ*^2^ tests. Statistically significant heterogeneity was defined as *p*-value < 0.1 for the *χ*^2^ test of heterogeneity. The *I*^2^ test was used to quantify the magnitude of heterogeneity, with 25%, 50%, and 75% representing low, medium, and high levels of heterogeneity according to the Methodology of Cochrane Handbook of Systematic reviews and meta-analysis, depending on the degree of heterogeneity [9].

## Results

**Fig. 1:**
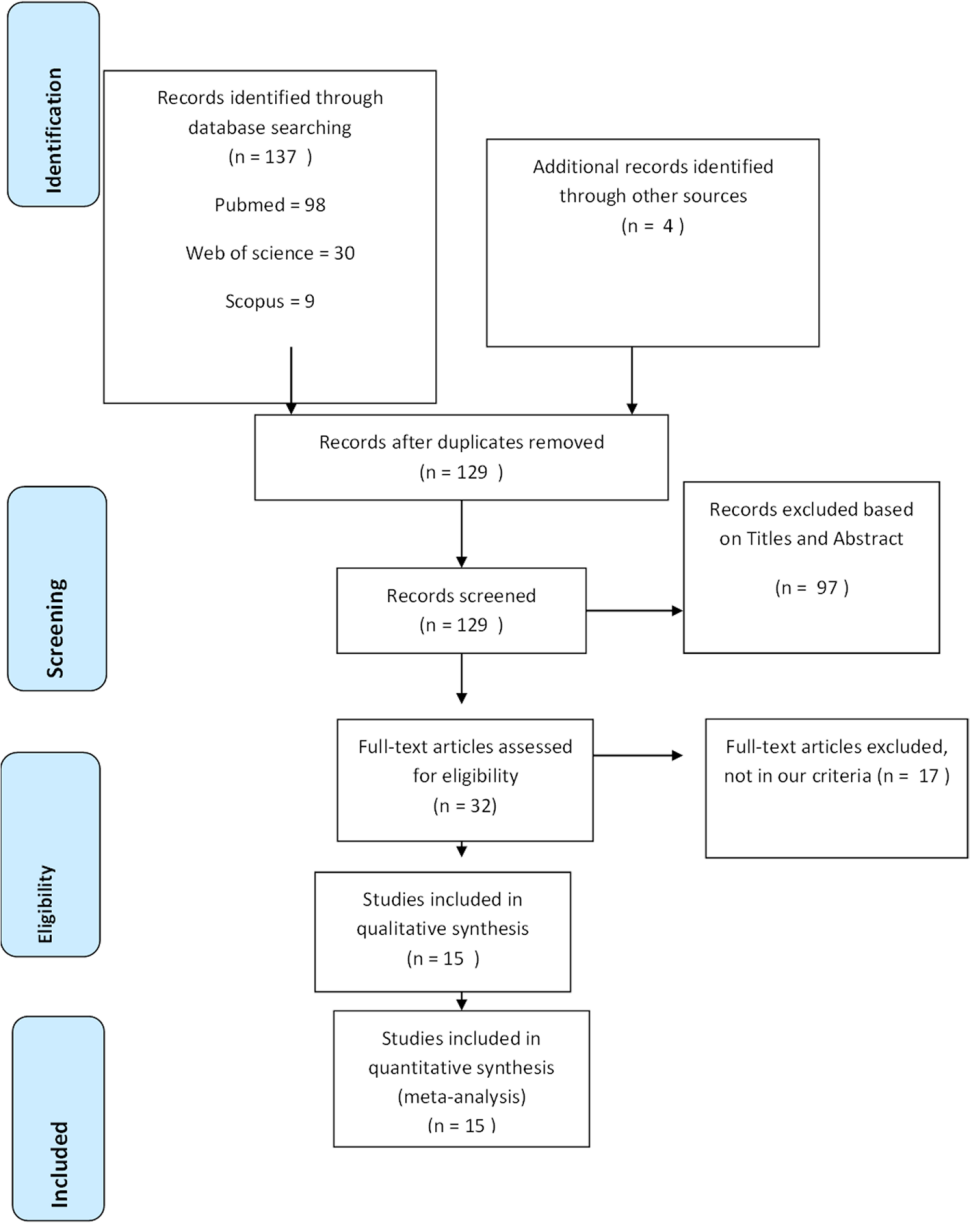
PRISMA flow diagram summarizing the literature search. PRISMA Preferred Reporting Items for Systematic Reviews and Meta-analyses

Details of our literature search are summarized in the PRISMA flow diagram (Fig. 1). Initially, 137 articles were identified and four articles were manually added after reviewing the reference lists. One hundred twenty-nine records remained after duplicates were removed, and 97 were excluded after reviewing their titles and abstracts. The full text of the remaining 32 articles was reviewed. Of these 32 articles, 14 were additionally excluded for the following reasons: two articles were review [10, 11], and twelve mentioned liver injury with COVID-19 but did not directly compare between the liver outcome in severe and non-severe COVID-19 cases. The most common definition of severe COVID-19 was based on clinical criteria, death, or ICU.

### Quality assessment

Overall, 12 studies were considered high quality, while three were considered medium quality (Table 1).

**Table 1:** Quality assessment

Author name	Selection		Ascertainment		Outcome		Overall
	Sample size adequate	Population representative (multicenter)	Test adequate	Comorbidity confirmation adequate	Outcome reported adequately (clinical staff)	Follow-up long enough (≥2 weeks)	Overall (≥4 stars = lower risk of bias)
Cai et al. [12]	*		*			*	***
Chen et al. [13]	*		*	*	*	*	*****
Guan et al. [14]	*	*	*	*	*	*	******
Huang et al. [15]	*		*	*	*		****
Lagana et al. [16]	*		*	*	*		****
Shi et al. [17]	*	*	*	*	*	*	******
Sonzogni et al. [18]	*	*	*	*	*	*	*****
Tian et al. [19]			*	*	*	*	****
Wander et al. [20]			*		*		**
Xie et al. [21]	*			*	*	*	****
Xu et al. [22]			*		*		**
Xu et al. [23]			*	*	*	*	****
Yang et al. [24]	*		*	*	*	*	*****
Zhang et al. [25]	*	*				*	***
Zhao et al. [26]	*		*		*	*	****

**Fig. 2:**
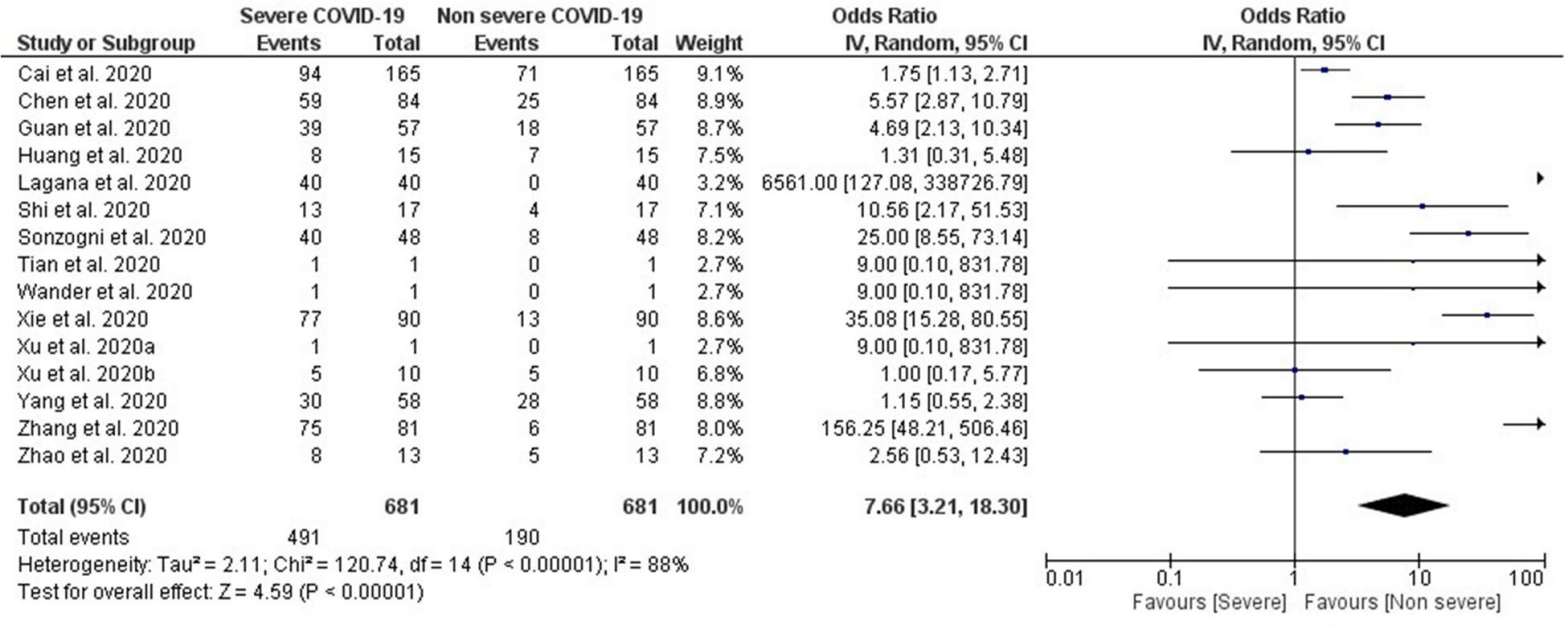
Forest plot showing the association between severity of COVID-19 and liver injury

Fifteen studies finally included in the meta-analysis (Table 2) showed that patients with underlying abnormal liver function and/or histopathological finding had a statistically significant 8.08 times higher odds of severe COVID-19 outcomes when pooling data from individual studies (*OR* 7.66; 95% *CI*, 3.21, 18.30; *p* = 0.00001) (Fig. 2).

**Table 2:** Liver outcome in severe and non-severe COVID-19 patients

	Sample size	Number of patients with abnormal liver function			Patients with pre-existing liver conditions			Positive liver histopathology findings		
		Total patients	Non-severe COVID	Severe COVID	Total patients	Non-severe COVID	Severe COVID	Total	Non-severe	Severe
Cai et al. [12]	417	318 (76.3%)	233 (71.5%)	85 (93.4%)	21 (5%)	nd	nd	nd		
Chen et al. [13]	274	84 (31)	25 (16)	59 (52%)	nd			nd		
Guan et al. [14]	1099	168/77 (22.2%)	112/615 (18.2%)	56/142 (39.4%)	23 (2.1)	22 (2.4%)	1 (0.6)	nd		
Huang et al. [15]	41	15 (31%)	7/25 (25%)	8/13 (62%)	1 (2.0%)	1 (4%)	0	nd		
Lagana et al. [16]	40	40 (100%)		40 (100%)	2/16 (13%)		2/16 (13%)	40 100%		40 100%
Shi et al. [17]	81	43 (53%)	4 (27%)	13 (62%)	7 (9%)	0	2 (10%)	nd		
Sonzogni et al. [18]	48	(40/41) 97.6%		(40/4) 97.6%	nd			48 100%		48 100%
Tian et al. [19]	4	1	0	1	1	0	1	1 25%	0	1 100%
Wander et al. [20]	1	1		1	nd			nd		
Xie et al. [21]	79	35%	13%	77.80%	nd			nd		
Xu et al. [22]	1	1	0	1	nd			1 100%	0	1 100%
Xu et al. [23]	62	10 (16%)	5 (17%)	5(15%)	7 (11)	3 (10%)	4 (12%)	nd		
Yang et al. [24]	52	15 (29%)	28%	30%	nd	nd	nd	nd		
Zhang et al. [25]	645	81	6 (8.3%)	75 (13.1%)	25	2 (2.8%)	23 (4.0%)	nd		
Zhao et al. [26]	91	18 (19.8%)	5/61 (16.4%)	8/30 (26.7%)	nd			nd		

*nd* no data available

Five studies (97 patients) showed histopathological changes at autopsy from cases with severe COVID-19: Lagana et al. [16], Sonzogni et al. [18], Tian et al. [19], Xu et al. [22], and Yao et al. [27]. In four of these five studies, the histopathology was associated with a history of abnormal liver function after infection with COVID-19: Lagana et al. [16], Sonzogni et al. [18], Tian et al. [19], and Xu et al. [22], but no data was available about liver enzymes in the fifth study, Yao et al. [27].

Lagana et al. [16] reported that patients dying from COVID-19 were found to have biochemical evidence of hepatitis (of variable severity) and demonstrated histologic findings with macrovesicular steatosis, the most common finding, involving 30 patients (75%). Mild lobular necroinflammation and portal inflammation were present in 20 cases each (50%). Vascular pathology, including sinusoidal microthrombi, was infrequent and was detected in six cases (15%). Only a total of 2 of the 40 patients had ballooning and Mallory–Denk bodies, indicating alcoholic or nonalcoholic steatohepatitis. PCR of liver tissue was positive in 11 of 20 patients tested (55%). They also identified viral RNA in liver tissue samples.

In another study [18] which described vascular changes, portal fibrosis was detected in 29 patients (60%), and an incomplete fibrous septum was seen in 8 patients (16%).

## Discussion

COVID-19-associated liver injury is defined as any liver damage in COVID-19 patients, whether or not they have pre-existing liver disease [28].

In our meta-analysis, cases with abnormal liver function and histopathological findings had a statistically significant 8.08 times higher odds of severe COVID-19 outcomes. In previous studies, the prevalence of elevated liver function tests (LFTs) in patients appears to be high [23]. There was variation in the prevalence of elevated liver function tests (LFTs) in patients with COVID-19 at admission due to the diversities of high LFT cut-off values and population variables [29].

Similar results to the current results were reported in a previous meta-analysis [10], where severe COVID-19 cases were found to be more associated with liver injury. Also, they found that the degree of liver injury was associated with the severity of COVID-19.

Our results were consistent with current literature that adult patients with severe COVID-19 have a higher risk of liver injury [11].

A potential cause of the hepatic injury may be the systemic effects of COVID-19. It is known that SARS-Cov-2 infects the pulmonary system, causing hypoxia and even acute respiratory distress, sepsis, and failure of multiple organs [15, 30]. COVID-19 sepsis leads to hypoxic damage and liver ischemia. It elevates liver biochemistries, demonstrating why total serum bilirubin, AST, and ALT levels are higher in severe COVID-19 patients than non-severe patients, as illustrated in our paper [31].

Our study reveals a coherent relationship between COVID-19 severity and liver injury, and hepatic injury mechanisms are uncertain and of multifactorial origin. SARS-CoV-2 direct damage (e.g., hepatocyte apoptosis) is proposed as a potential mechanism [32]. The expression of ACE2 protein (the SARS-CoV-2 entry receptor) on cholangiocytes (bile duct cells) supports the virus-mediated liver damage possibility. Cholangiocytes are more than 20-fold expressed by ACE2 receptors than hepatocytes. Although cell damage can occur at the level of bile ducts, the trends show an elevation in amino-transferases rather than gamma-glutamyl and alkaline phosphate [33, 34]. Therefore, it is crucial to formulate various mechanisms. A deliberation should also be made about drug-induced liver injury in addition to direct viral-mediated damage [35]. Moreover, some antiviral drugs can be hepatotoxic concurrently in these patients [36].

Underlying liver comorbidity, one of the most critical forecast variables, has been recognized for deterioration due to COVID-19 [29]. This finding is inconsistent with other cohorts [28]. In the previous systematic review and meta-analysis, patients with the previous liver disease did not appear to be at increased risk for disease progression [37]. Further studies are needed to analyze the outcomes in COVID-19 patients with and without liver disease.

Of note, our study observed that the severity of COVID-19 was associated with more patients with aberrant liver function tests. Some studies have also shown a definitive prognosis of COVID-19 is related to aberrant LFTs. For example, 16 retrospective cohort studies in China were recently analyzed, summarizing higher levels of markers of hepatic injury, especially transaminases, with increased morbidity and mortality [38]. Our findings could encourage further studies in this area in order to identify mechanisms that cause liver damage.

Lagana et al. [16] reported that macrovesicular steatosis was common (75%), with fat distribution not typical of NAFLD. Features of frank steatohepatitis were present in only two cases. Therefore, it seems likely that the steatosis in some of these cases may have developed during the course of their COVID-19 illness. This finding is consistent with other studies; for example, a study performed by a working group affiliated with the Centers for Disease Control found steatosis in 50% of autopsy livers [39].

Lagana et al. [16] explained the etiology may be multifactorial with corticosteroid administration, hypoxia, malnutrition, and direct viral effects all plausible considerations (although neither corticosteroid use nor length of stay, a surrogate for malnutrition, was statistically associated with the amount or distribution of steatosis in their study). A series of more than 300 COVID-19 cases in China investigated the relationship between steatosis and the neutrophil to lymphocyte ratio (NLR). A higher ratio suggests more severe systemic inflammation. This study showed that steatosis and NLR were associated with the severity of illness so that steatosis and high NLR can predict more severe cases [40].

They [16] found lobular necroinflammation in 50% of cases. Infrequent hepatocyte apoptosis was observed. In addition, mild but not moderate necroinflammatory activity was observed in four cases. Necroinflammatory foci consist of one to several dead and dying hepatocytes with few accompanying lymphocytes and histiocytes. Plasma cells have been rarely encountered. They reported that the cohort of histologically defined acute hepatitis cases did not demonstrate higher liver enzyme levels compared to the cohort without lobular necroinflammation.

The ACE2 receptor is present on cholangiocytes [41], but bile duct injury has not been observed. However, cholestasis was common (38%) [16].

The histopathological findings of another study [18] with marked derangement of the intrahepatic blood vessel network are highly suggestive of systemic changes induced by the virus. Portal fibrosis was seen in 29 (60%), and incomplete fibrous septa were detected in 8 (16%). Histological findings strengthen the hypothesis that the derangement of the coagulation process and impairment of blood circulation inside blood vessels or endothelial damage could be the mechanism in the pathogenesis of COVID-19 damage [6]. A diffuse network of sinusoids with positive immunoexpression of CD34 suggests a disturbed circulation of blood within the liver [42].

### Limitations

The current study has a number of limitations. First, patients may often not be conscious of underlying chronic liver disease (CLDs) (e.g., fatty liver diseases). Therefore, they can be mistaken for not having CLD. This may result in the underestimation of baseline CLD prevalence. Second, as previously illustrated, our study measurements were substantially heterogeneous. This is possible because small-scale research of varying prevalence is often included. Finally, all the studies investigated were observer-based, and this feature of COVID-19 must be randomized. While a meta-analysis can improve competence and give better estimates, the current findings are systematic to provide early intuition and should not be viewed as an alternative to large-scale observational studies.

This study analyzed histopathological findings at autopsy, which means that it describes patients with severe infection. As the epidemic evolves, it will be important to collect data on liver injury in patients with non-lethal COVID-19. Morphological studies describing liver parenchymal changes induced or related to SARS-CoV-2 infection are lacking. In the present, only post-mortem reports are available.

## Conclusions

This systemic review study and meta-analysis show that a higher occurrence of liver injury has been consorted with COVID-19. Moreover, the severity of the disarray in serum liver function indicators is linked to higher acuity. Thus, special attention should be given to any liver dysfunction while treating patients with COVID-19.

## Data Availability

All data used in this paper are available for consultation after a suitable request from the corresponding author.

## Abbreviations

ACE: Angiotensin-converting enzyme
CD34: Cluster of differentiation 34
CLDs: Chronic liver disease
COVID-19: Coronavirus disease 2019
LFTs: Liver function tests
NAFLD: Non-alcoholic fatty liver disease
NLR: Neutrophil to lymphocyte ratio
PCR: Polymerase chain reaction
RNA: Ribonucleic acid
SARS-CoV-2: Severe acute respiratory syndrome coronavirus 2
